# Students’ perceptions on community-based education at Avalon University School of Medicine during the first two years of the program

**DOI:** 10.15694/mep.2018.0000190.1

**Published:** 2018-09-04

**Authors:** Sateesh Babu Arja, Sireesha Bala Arja, Venkata Anirudh Chunchu, Narendra Sai Varma Datla, Archana Bottu

**Affiliations:** 1 Avalon University School of Medicine

**Keywords:** clinical skills, community services, communication skills, community-based education, and experiential learning.

## Abstract

This article was migrated. The article was not marked as recommended.

Objectives

The socio-cultural learning theory can be divided into a social and cultural process. Learner’s learning occurs within the context rather than anticipating and preparing for the future context. It may happen in the workplace through apprenticeship, experiential learning, or community-based learning. Community-based education and community services have always been part of the students’ volunteer services at Avalon University School of Medicine. The importance of community-based education has led to its recent integration into the curriculum. The objective of this study is to observe the community services in the field and to record the perceptions of students regarding community-based education at Avalon University School of Medicine.

Methods

This is a qualitative study. The research was conducted in the form of an observational study and framework analysis was done. The community-based education and community services were observed and recorded along with individual interviews. Students from different semesters were selected randomly for the interviews. The interviews were audio-recorded and transcribed.

Results

All interviewed students (100%) reported that they are involved in community services. 53.8% of students were not able to recognize the health issues of Curacao. 84.6% of students recognized and acknowledged the local health issues after reminding them of the activities conducted in the community services. 84.6% of students believed community services enhanced their clinical skills and increased their confidence in communication skills.

Conclusion

Community-based education enhances the competency of future physicians in clinical and communication skills.

## Introduction

One of the common problems that are encountered during the training of junior doctors and post graduation programs is that the doctors struggle with advising patients in a local socio-cultural context. These issues may stem from miscommunication or a lack of understanding the local culture. The community-based education enables medical students to understand the societal needs and social factors influencing the health and illnesses. Participation in outside-classroom teaching and involvement in community services with the population who lacks health literacy in the pre-clerkship years increases the confidence and better communication skills ( Milford et al., 2016).

The current medical profession and healthcare services face a disparity in physician distribution and healthcare services between rural and urban areas. Early exposure to the community in medical programs and involvement in the rural community may motivate medical students to practice in rural areas ( Budhathoki et al., 2017). The community-based education in the undergraduate medical curriculum also promotes the contribution to the local health services and aids the community health needs ( Atuyambe et al., 2016).

Talaat and Ladhani provide a concise historical account of CBE in the health professions ( Talaat and Ladhani, 2014). They state that: Community Based Education (CBE) is a form of instruction in which students learn professional competencies in a community setting, helping to build a sense of connection with their communities. CBE is a popular approach for all forms of education and for all age groups, especially at higher education levels where its primary purpose is to foster interdependence between education and communities, leading to improved quality of life. John Hopkin’s definition of Community Based Learning (CBL) Working Group states “it is a pedagogical model that connects classroom-based work with meaningful community involvement and exchange.”

Medical students should start understanding patients’ cultures and beliefs related to their health and illnesses from pre-clinical years. The understanding should be encouraged through the curriculum and planned learning experiences. The curriculum should offer opportunities for experiential learning. Community-based education teaches and trains students to understand the social and cultural context of patients’ principles regarding the health beliefs.

Understanding of societal factors should be integrated into the curriculum in the form of both theoretical knowledge and practical learning opportunities. Students should have service-learning experiences throughout the curriculum. The students should be informed about the community strengths and the population health aspects of the community, too. Service-learning is a teaching and learning strategy that integrates meaningful community service with instruction. It enriches the learning experience, teaches civic responsibility, and strengthens communities, according to the National Service-Learning Clearinghouse’s definition (Service learning, 2012).

Learning in the community is as effective as learning in the clinical skills labs and hospital systems ( Murray, Jolly and Modell, 1997). Community-based education also fosters experiential learning. This experiential learning can be related to Kolb’s experiential learning ( Kolb, 1984; Kolb and Kolb, 2005).

**Figure 1.**
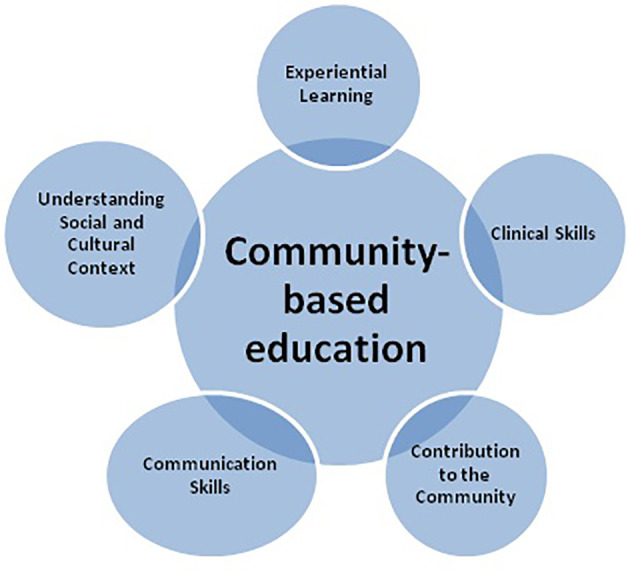
Beneficial effects of community-based education and activities.

The below figure 1 summarizes of benefits of community-based education.

### Background

Avalon University School of Medicine was established in 2003 on the island of Bonaire. The university relocated to Curacao in 2010. The community-based education and community services have been a part of Avalon University School of Medicine since its inception. The curriculum of Avalon University School of Medicine has been recently reviewed and changed from a discipline-based curriculum to the organ-based curriculum. During the process of the change, the community-based education and service learning has become a mandatory part of the curriculum. The university realized the importance of community-based education for the reasons mentioned above in addition to the recognition of social accountability of the educational institutions ( Boelen and Heck, 1995; Boelen and Woollard, 2009; Global Consensus for Social Accountability of Medical Schools, 2010).

Community-based education and community services are part of the curriculum and are included in the Clinical Skills course. The Clinical Skills course is placed longitudinally throughout all semesters of the basic sciences program. The curriculum committee placed the community-based education longitudinally along with clinical skills. Longitudinal experiences are more beneficial than snap-shot learning experiences. The university developed and adopted a basic framework to carry out community-based education in the first two years of the program.

**Figure 2.**
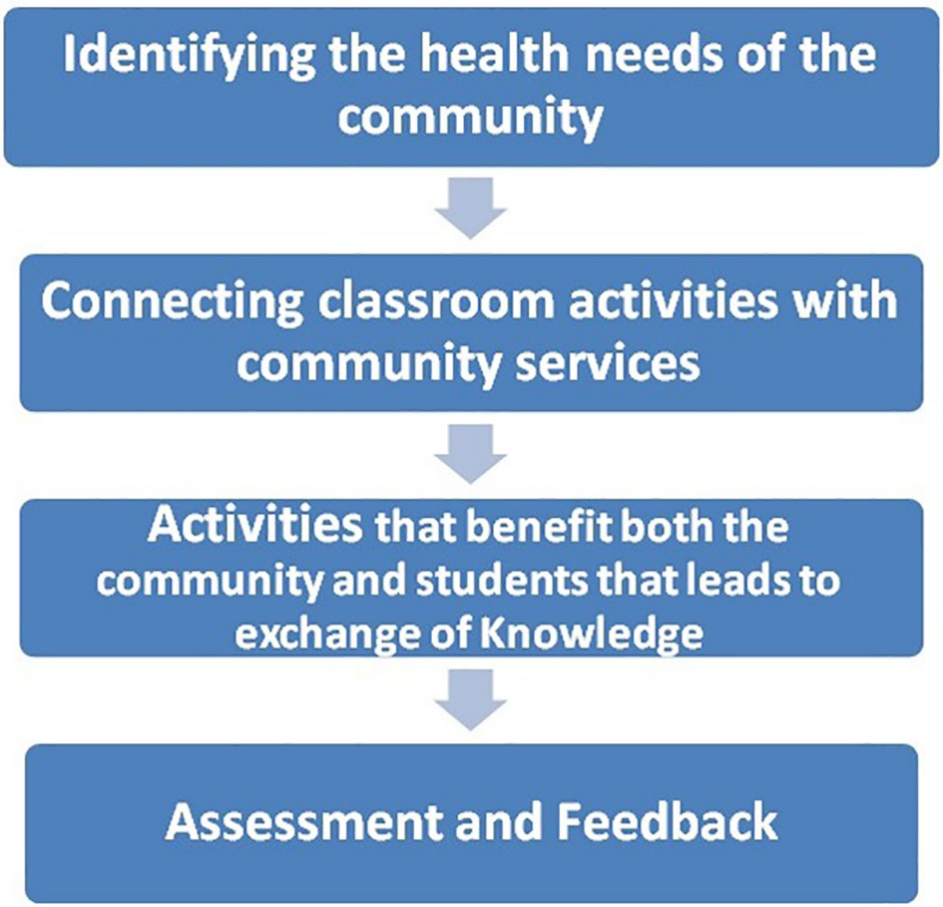
Framework adopted by the University for community-based Activities and education

## Methods

This research study is a qualitative and framework analysis was done. An observational study was conducted to analyze the activities involved in the community-based education and community services and the data was recorded. Pictures were taken while observing the activities. Consent was taken for all identifiable persons in the pictures. Students from the second-semester and the fourth-semester were interviewed individually. Consent was taken from all participants and it was informed that interviews will be kept anonymous. Student interviews are numbered as student #1, student# 2 and no personal information is identified. Participation in the interviews was voluntary and all students were given the option of not to participate. All individual interviews were audio-recorded and later on, they were transcribed.

**Table 1.** Student Demographics of the Second and fourth semester at Avalon University School of Medicine

		Biologic Sex		Race or Ethnicity			
Cohort	Total #	Female	Male	Caucasian	Hispanic	African	Asian
2 ^nd^ Semester	11	6	5	0	1	2	8
4 ^th^ Semester	21	9	12	2	0	2	17

Student selection for the interview process was randomized instead of the purposive sampling. Every third student in the fourth-semester was interviewed based on alphabetical order. Seven students (three male and four female) were interviewed. Among the second-semester students, we skipped selecting the first student on the alphabetical order and then interviewed every alternative student. Students in alphabetical order interviewed are 2 ^nd^, 3 ^rd^, 5 ^th^, 7 ^th^, 9 ^th^, and 11 ^th^ student (3 male and 3 female students). Students of second-semester are interviewed by the first-year medical student and students of fourth-semester are interviewed by the second year medical students. The interviewed students are diverse and representative of the total Avalon University School of Medicine population.

## Results/Analysis

### Observational Study

#### Identifying the health needs of the community

The medical school identified the health needs of the community to include overweight/obesity, diabetes, and hypertension. The identification was based on the data released by National Health Survey 2017, a survey released by the Ministry of Health, Curacao (Curacao Chronicle, 29 ^th^ January 2018). According to the survey, 65% of the island population is either overweight or obese. Being overweight or obese is the predisposing factors to hypertension and diabetes. The factors leading to being overweight or obese are including, but not limited to, genetic factors, sedentary lifestyles, and dietary habits.

#### Connecting classroom activities with the community services

Students are trained on how to measure the blood pressure, body mass index, and blood glucose in the classroom. Students are also trained on history taking and communication skills in the Clinical Skills course. In addition to classroom training, students are trained in practice sessions before each community activity. We observed three of these training sessions. Students are divided into groups and trained on these activities in a professional manner. Students practice these skills on standardized patients.

**Figure 3.**
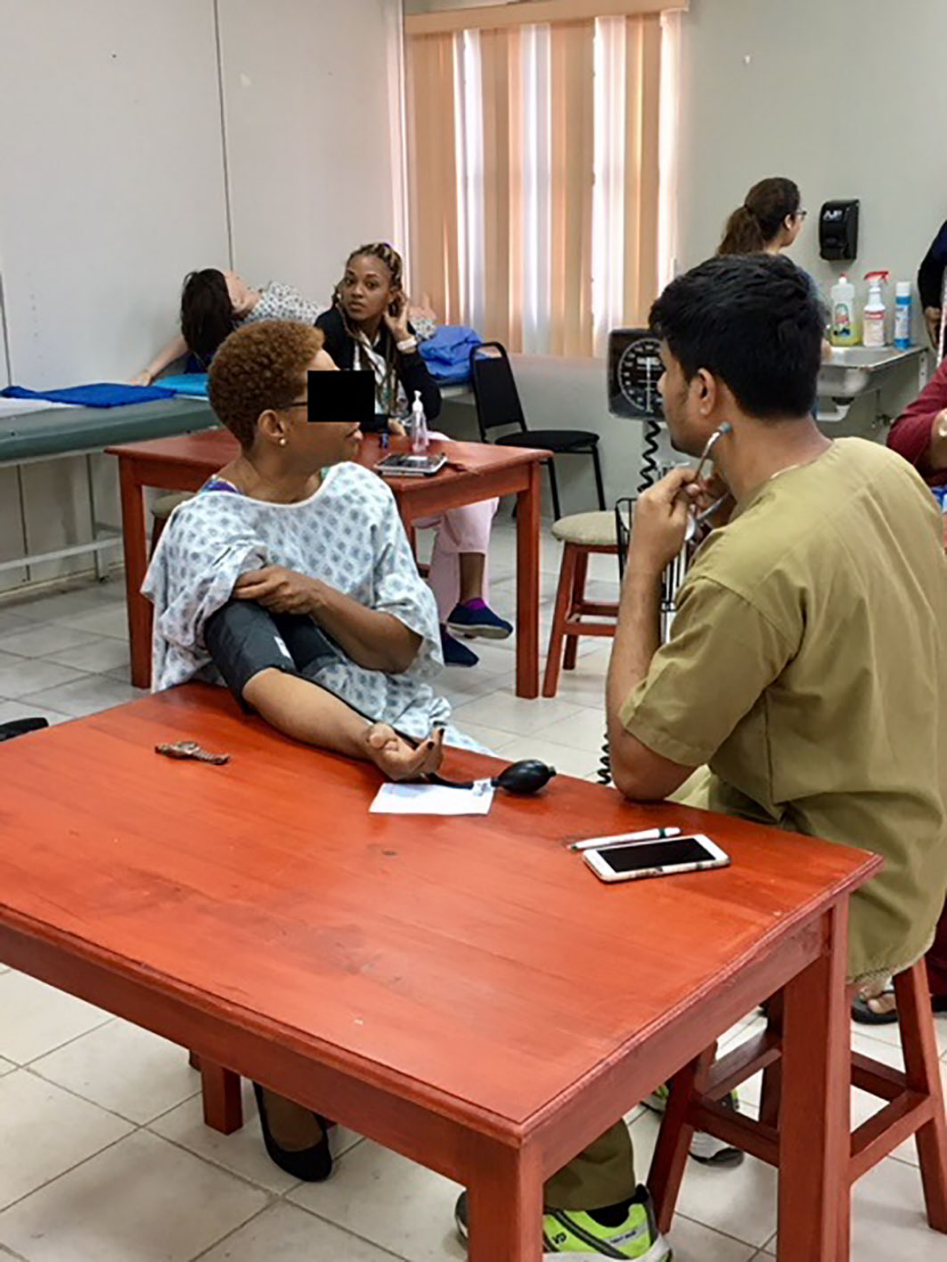
Students getting trained inside the classroom on standardized patients

#### Students Involvement in Community Activities and Community Benefits

We observed students of Avalon University School of Medicine involving themselves in community activities several times. The services provided by Avalon students are: screening the public for body mass index, blood glucose, and blood pressure. The population was counseled regarding lifestyle, diabetes, hypertension, obesity, and modifiable risk factors. This provided an opportunity for the students to understand the societal needs and healthcare issues. Students build communication skills, professionalism, and leadership skills; priming to become better physicians. This also fulfills the social responsibility and social accountability of the medical school towards the community and local health need to some extent.

**Figure 4.**
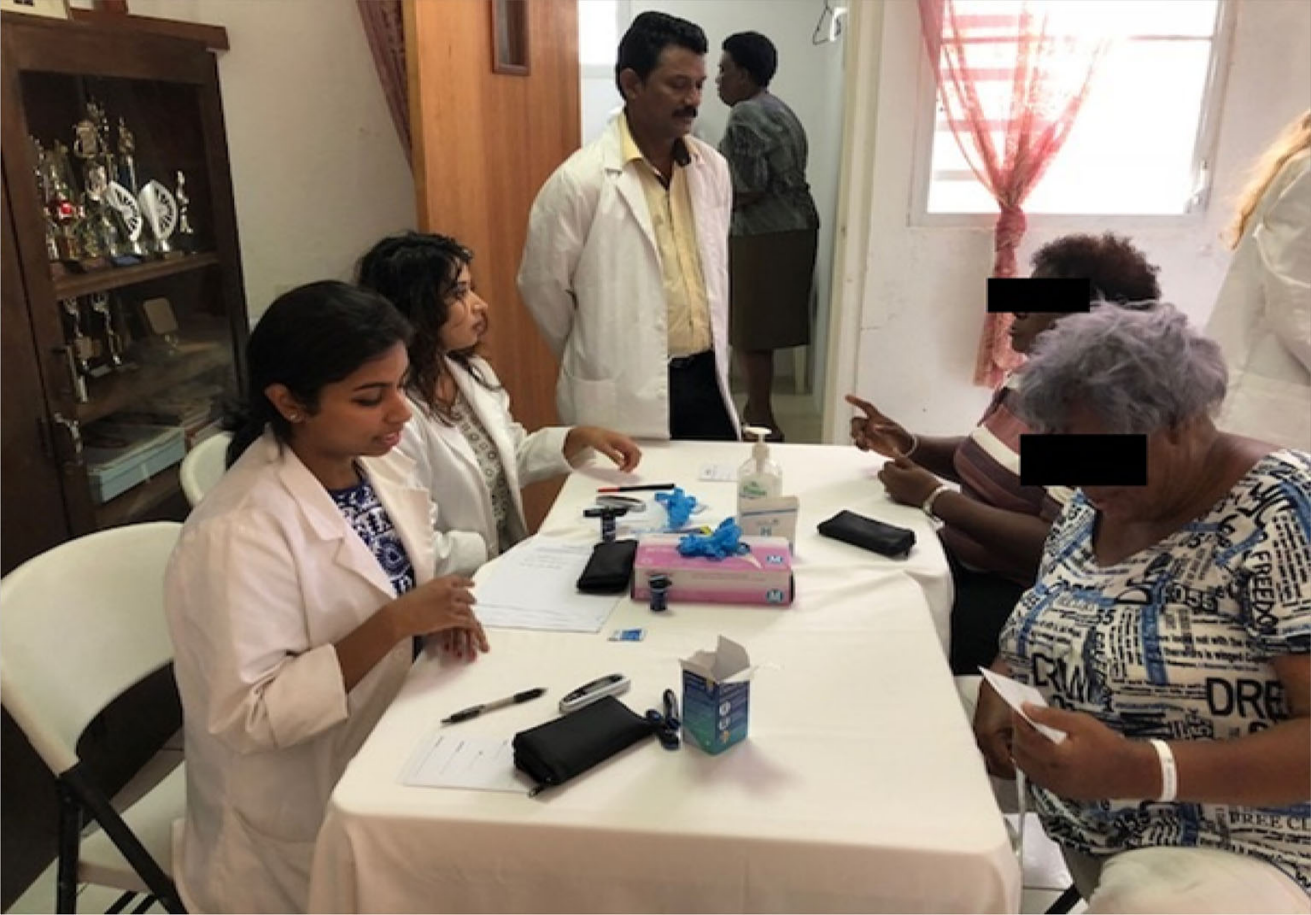
Students involved in community services

**Figure 5.**
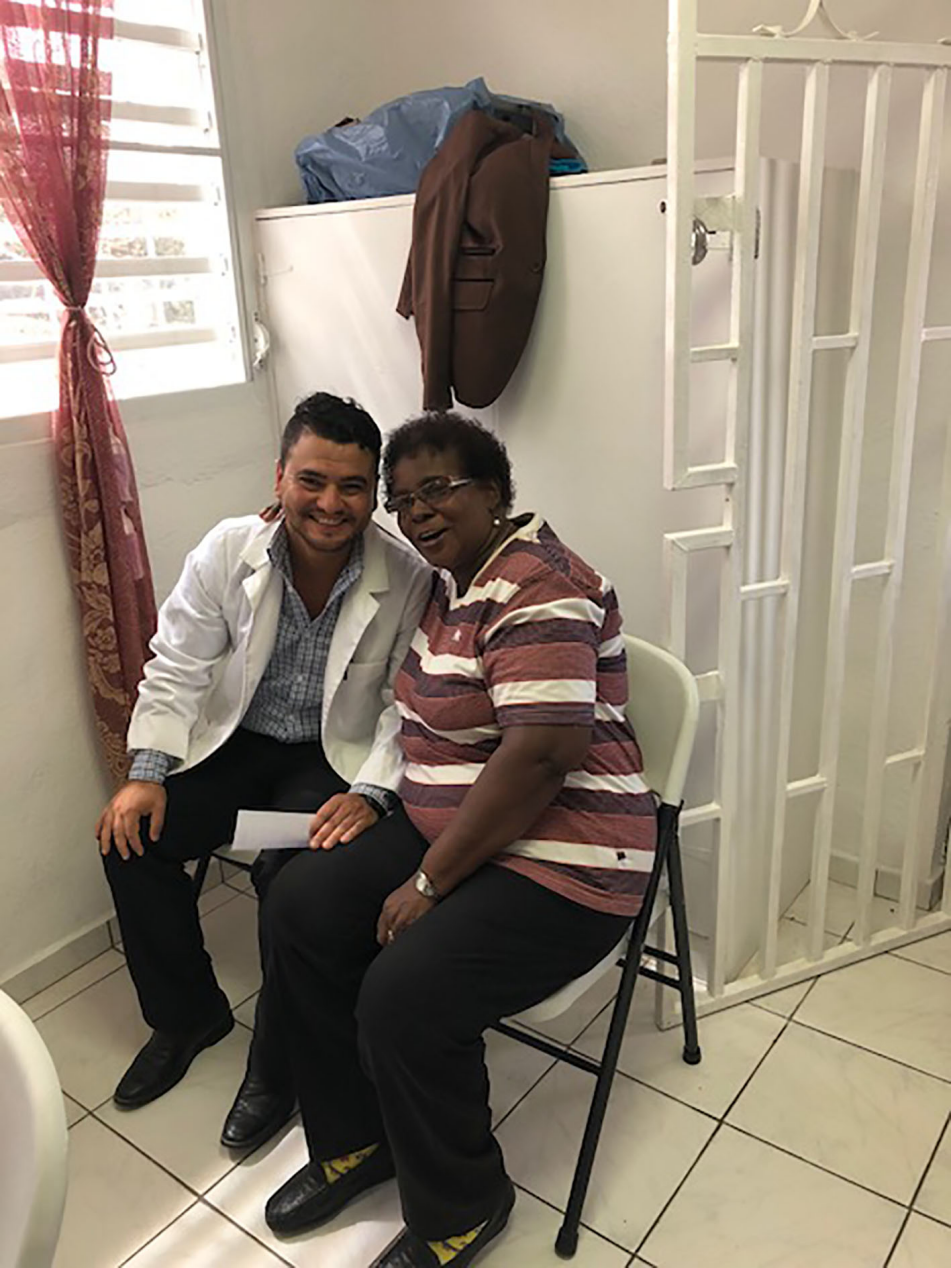
Happy community member posing with one of the students of Avalon University School of Medicine

#### Individual student interviews

A total of 13 Students were interviewed individually and the following questionnaire was used. The questionnaire was created based on the framework developed by the university regarding community-based education. The questions are either yes/no questions or open-ended questions. Students were asked about their experiences and were encouraged to give descriptive answers.

• Have you ever participated in community services at Avalon?
• Do you know the health issues of Curacao? If yes, please explain.
• You are doing BMI, and measuring blood pressure and blood glucose. The health issues of Curacao community include overweight/ obesity, diabetes, and hypertension. Do you recognize them and relate with your activities in the community? This question was included to see if students can recall the health needs of the community.
• Do you think that you were trained in the classroom on how to measure BMI, blood glucose, and blood pressure? Please explain.
• Have you ever thought that participating in community services increase your clinical skills and enhance the confidence in communication skills and advising the patients in the local socio-cultural context? Please describe your experiences.
• Are you getting a grade or assessed based on the involvement in community services?
• Do you need a feedback after every community activity? Please comment on how you want to receive the feedback and what kind of feedback are you expecting?

Student responses are tabulated ( Table 2).

**Table 2.** Students’ responses collected through individual interviews.

Student Number	1	2	3	4	5	6	7	8	9	10	11	12	13
Participation in Community Services	Yes	Yes	Yes	Yes	Yes	Yes	Yes	Yes	Yes	Yes	Yes	Yes	Yes
Aware of Health issues of Curacao	Yes	Yes	Yes	Yes	No	No	Yes	No	No	Yes	No	No	No
Recognized the Health Issues After Reminding	Yes	Yes	Yes	Yes	Yes	Yes	Yes	No	No	Yes	Yes	Yes	Yes
Classroom Activities and Training	Yes	Yes	Yes	Yes	Yes	Yes	Yes	Yes	Yes	Yes	No	Yes	Yes
Positive Perception of Community activities	Yes	Y/N	Yes	Yes	Yes	Yes	Yes	Yes	No	Yes	Yes	Yes	Yes
Assessment	Yes	Yes	Yes	Yes	Yes	Yes	Yes	Yes	No	Yes	Yes	Yes	Yes
Do the Students Need Feedback?	No	Yes	No	Yes	Yes	Yes	No	No	No	Yes	Yes	Yes	Yes

## Discussion

Students’ responses were analyzed. 100% of the students responded that they were involved in community services and community-based education. Seven out of thirteen students (53.8%) answered that they were not aware of the health issues in Curacao, even though some of them acknowledged the health issues after a reminder of the health issues they encountered while interacting with the local communities. It is required by the instructors to inform the students why the university has chosen certain activities: this community-based education/activity must address the local health issues as part of the social responsibility. Eleven out of thirteen students (84.6%) recognized and acknowledged the health issues of the island after reminding them what they have done during the community services.

Twelve out of thirteen (92.3%) students recognized that they were trained, and classroom activities related to community-based activities. Eleven out of thirteen students (84.6%) felt that community-based education and community activities enhance their clinical skills and improve their overall communication skills. The following are quotes of the students:

“ *It’s the biggest part of being a doctor, being able to be part of the community and help in achieving the community health goals.*”

“You go out and actually meet people at random and help with actual health problems. For example, the first time I ran into this lady that had diabetes, she was not looking after herself at all. The same thing happened with the second activity we did. We had this patient, with the edema of her foot. I don’t know if they are actually following advice and going to their doctors after that, but at least you can see that there are things that you can identify for them. Let them know they have something and need treatment or let them at least know they have something they need to pay attention to.”

“ *Working with standardized patients in clinical skills is entirely different. We talk to the standardized patients all the time and become our friends. We can be however we want, act however we want. And they give us pointers. We see them all the time. Talking to somebody out there, especially when they don’t know English, where you have to translate, and do everything, and think on your feet is a lot different. I think that helps a lot. It gives us a chance to interact with patients and regular people you don’t get to see. It makes you think on your feet. You get to see regular patients, which we don’t always get a chance to see in the basic sciences.*”

“I personally really enjoyed it. I think we should be doing more things like that. It gives the students an overall image of where they’re heading. It gives overall motivation, which is important in such a fast-paced program like this. It is something that is really important because you need to compound on your skills and get better”.

“ *In a classroom, you are with people that you are comfortable with like your classmates. Outside, you have to actually describe what you are doing, that could be hard to practice on.. One thing I noticed from the community activity that we did: there was a lot of noise in the background. You were not actually able to hear the tick to calculate the blood pressure. So being outside in the real-world helps in developing your skills.. Even in hospitals, sometimes things could happen. So, if you are prepared for any sort of situation and I think community services help with that.*”

“It helped to understand the local culture. These services taught me the importance of local culture and working within the cultural context.”

“ *Aside from studying and books, it’s hands on. Instead of working and books, you get to see what you’ve signed up to do for the rest of your life.*”

As mentioned in table 2, two out of thirteen students were not positive about community services really can help with improving communication skills or not? One student was equivocal about community services. Their main concern is the language barrier, even though most of the population on the island speaks English. Here are their comments in their own words:

“Community services do and it doesn’t help with communication because we aren’t speaking the native language. So, we can’t really communicate properly with patients i.e. residents that come to us at the community services. But it is a good way to enhance and teach us how to talk to people that don’t necessarily speak the native language, which should be useful in the hospital that teaches us on how to take blood pressure and comfort the patient in a way and build up our skills as training physicians on how to take vitals”.

“To be honest, for me, no. Because you know especially on this island, their primary language is very different from mine, so I don’t feel it enhances my communication skills. Again, not because there is a strong language barrier”

Regarding the assessment, twelve out of thirteen students (92.3%) felt that they were assessed based on the community services and they were given a grade based on their involvement in the activities. Regarding the feedback, seven out of thirteen students (53.8%) would like to have a feedback regarding their performance in community activities. But the other six students felt that it is not necessary for them.

Here are some comments from students in their own words regarding feedback:

“I guess I would want it incorporated in class, like the same way in what we do in clinical skills practice. I would like the teacher to come to me and say these are the negatives and positives. These are the things that have to change. I would just want them to be honest and direct.”

“I don’t think feedback can hurt. That’s for sure. I’m always looking to improve or learn or anything like that. If I’m doing something wrong or could be done better. Forget about doing anything wrong. Even if it’s doing better, I’d be open to it”.

“I would say maybe through email if you have a report or maybe you as a professor call them as students and ask questions and maybe discuss with them”.

## Conclusion

Based on the results, students appreciate community-based education and involvement in community services. The early exposure of medical students to the community and general population is crucial in enhancing their clinical skills, communication skills, and building their confidence as future physicians. Even though the language barrier may limit some aspects of community-based education, translators are a viable solution. At our university, this problem can be easily solved because: the majority of the population can speak English, and our standardized patients are locals that can aid in translation.

## Take Home Messages

• Involving in community-based education (CBE) enhances clinical skills as they are exposed to the real world. CBE can be helpful, especially in circumstances where students are not exposed to real patients, aside from standardized patients in the first two years of the medical program.
• CBE helps in developing communication skills and fostering the confidence of future physicians.
• CBE can help in understanding the societal factors and working in the cultural context.
• Translators can be helpful if there are any language barriers to conduct community-based activities.
• Assessment and feedback should be part of the community-based education.

## Notes On Contributors

Dr. Sateesh Arja, MD, MPH, SFHEA, FAcadMEd, AFAMEE, Director of Clinical Skills course and Executive Dean.

Dr. Sireesha Bala Arja. Associate Professor of Pharmacology, and Associate Dean of Basic Sciences.

Mr. Venkata Anirudh Chunchu, 2nd-year medical student.

Mr. Narendra Sai Varma Datla, 2nd-year medical student.

Ms. Archana Bottu, 1st-year medical student.
