# Supplementary material for: Students’ perceptions on community-based education at Avalon University School of Medicine during the first two years of the program
Source: MedEdPublish (2016). 2018 Sep 20;7:190. Originally published 2018 Sep 4. [Version 2] doi: 10.15694/mep.2018.0000190.2 (PMC10701824; doi:10.15694/mep.2018.0000190.2)
Supplement: Peer review history

# Reviewer report: Reviewer response for version 2

**DOI:** 10.21956/mep.20226.r31328

**Published:** 2019-04-05

**Authors:** Flora Smyth Zahra

**Affiliations:** 1 Faculty of Dentistry, Oral &Craniofacial Sciences. King's College London

This review has been migrated. The reviewer awarded 4 stars out of 5 I enjoyed this CBE paper very much and wish you well with your co-created programme. Your students obviously have benefited from the experience. It would be great if we all could drop the term standardised patient. I know its a very common term, but along with Human Factors skills and worse still 'soft skills' I wish we could work to improve them as they are all discordant to me in the context of person-centred care.

Reviewer Expertise:

NA

No decision status is available

# Reviewer report: Reviewer response for version 2

**DOI:** 10.21956/mep.20226.r31331

**Published:** 2018-10-24

**Authors:** Tharin Phenwan

**Affiliations:** 1 University of Dundee

This review has been migrated. The reviewer awarded 3 stars out of 5 This article assessed undergraduate medical students’ perception of Community-Based Education (CBE). There were gaps in the lack of understanding of the local contexts in the current medical curriculum. Thus, the authors proposed to use CBE approach to build a sense of connection to the community.-This second version of the revised manuscript was well-described. The authors wrote with a good narrative writing style with a clear background of the context of their medical school, their curriculum, and rationale to use CBE. -It is very pleasing to see that some medical students were involved in this work as the authors. This experience will be a good foundation for their research skills and enhance their profiles as well. -CBE plays a huge role in 21st-century learning skills, especially on Collaboration and Communication skills component. Practical assessment of this method will be useful in the education field.However,-How did the authors ensure the quality of the interviews? Was there any training session for them? -You mentioned that the participation was voluntary, but another paragraph stated that it was randomised by every third student. Could you clarify this? Did you mean by every third of the whole class or every third of the willing participants? The student selection for the interview part was also hard to follow with the current description. Another figure or diagram may help the readers grasp this more easily. -Most of the results section should be under the methods section as it will provide a clearer understanding to the readers. For example, the connecting classroom activities with the community services section was the description of how you prepared and validated the students before they went into the community. These descriptions were not what you did to the community but rather what you prepared before you took the students into the community.-The discussion section should focus more on the analysis and discussions of your findings, i.e. from these students’ comments, most of them had a positive experience about CBE. Why is that? Is it related to Kolb’s learning theory or others? -You can also select a few of the high-lighted quotations with important findings and put them into themes under one table to make it easier to follow-through. -This paper mentioned the cultural influence a few times. Could you add some examples of how culture would affect the patients and medical students in their learning experience and also the junior doctors? You can also add more reference from Hofstede’s work on six dimensions of national culture for the cultural framework.Overall, the authors used CBE to help medical students learn from the community and participants had a positive experience from this approach. Future works could follow this group of students to see their academic achievement. More works that compare between CBE and other learning methods of the students’ learning outcome are also recommended.This article is suitable for educators who emphasised on experiential learning and community-based learning despite some methodological limitations.

Reviewer Expertise:

NA

No decision status is available

# Reviewer report: Reviewer response for version 2

**DOI:** 10.21956/mep.20226.r31327

**Published:** 2018-10-15

**Authors:** Trevor Gibbs

**Affiliations:** 1 AMEE

This review has been migrated. The reviewer awarded 3 stars out of 5 This is the second version of this authors paper. The authors as before should be congratulated on the initiative in taking the students out of the medical school environment and placing them in the real world of patient care. I have no doubt that the students enjoyed this activity and it gave everything a sense of purpose. However I think that the authors do need to be cautious about what they can draw upon as a result of this activity and their point "Based on the results, students appreciate community-based education and involvement in community services." - taken from their text- is probably appropriate. I do not though feel that they can place great importance in their other Take Home Messages, given that this small number of students and the evaluation methodology chosen, does not really support these statements. I am very sure that this is a learning situation for all the authors and I wish them well in developing their community-based education- I approach I very much feel is important.

Reviewer Expertise:

NA

No decision status is available

# Reviewer report: Reviewer response for version 2

**DOI:** 10.21956/mep.20226.r31330

**Published:** 2018-10-01

**Authors:** Joy Cox

**Affiliations:** 1 American University of Antigua

This review has been migrated. The reviewer awarded 4 stars out of 5 Thank you for contributing to the literature on Caribbean medical education. Community-based education is indeed a teaching and learning strategy that integrates community service with instruction and is important in experiential learning for undergraduate medical students. The paper is well written, and the introduction provides the context for the study and the theoretical framework. The methods section is concisely written and clearly describes student selection. The results and discussion adequately refer back to the theoretical framework and I like the use of individual students quotes to substantiate how students perceive the benefits of service learning opportunities. One comment I have is that it is recommended that you place supplementary materials, such as the photos, in the appendix, and refer to them, so they will not interfere with the flow of the passage for the reader.

Reviewer Expertise:

NA

No decision status is available

# Reviewer report: Reviewer response for version 2

**DOI:** 10.21956/mep.20226.r31329

**Published:** 2018-09-23

**Authors:** P Ravi Shankar

**Affiliations:** 1 American International Medical University

This review has been migrated. The reviewer awarded 5 stars out of 5 I had reviewed an earlier version of the manuscript and the changes the authors have carried out in the revised version further strengthen the manuscript. The manuscript is well written and I congratulate the authors on their efforts at educational innovation and scholarship under the resource-constrained environment of a Caribbean medical school. Figure 4 seems to be distorted which can be corrected. I also am pleased by the fact that students are involved as coauthors in this research paper. The manuscript will be of interest to all medical educators and of special interest to those teaching in Caribbean offshore medical schools.

Reviewer Expertise:

NA

No decision status is available

# Reviewer report: Reviewer response for version 1

**DOI:** 10.21956/mep.19113.r27567

**Published:** 2018-09-07

**Authors:** P Ravi Shankar

**Affiliations:** 1 American International Medical University

This review has been migrated. The reviewer awarded 4 stars out of 5 I enjoyed reading this article about community-based learning (CBL) at an offshore Caribbean medical school. Caribbean offshore schools face challenges with community involvement and in integrating and working with local health systems. Health camps are commonly used by schools to introduce students to the local community. A brief description of the community-based activities and education at Avalon University will be helpful. How do the students gain practical experience in the community? Do they conduct health fairs and camps or do they visit the individuals in their homes? The authors mention mainly three activities measuring BMI, blood glucose and blood pressure as connecting classroom learning with community practice. How frequently do students participate in community learning activities? What exactly do students do during the community encounters? The pictures add to the description provided in the text. Community-based learning is becoming increasingly important for offshore schools which face some unique challenges in this regard. Some of the quotes from the students are better presented in the Results section. The authors have to be commended on their efforts and I look forward to further developments in CBL at their institution. .

Reviewer Expertise:

NA

No decision status is available

# Reviewer report: Reviewer response for version 1

**DOI:** 10.21956/mep.19113.r27568

**Published:** 2018-09-04

**Authors:** Trevor Gibbs

**Affiliations:** 1 AMEE

This review has been migrated. The reviewer awarded 2 stars out of 5 Community-based Education ( CBE) is an important element of the curriculum and has been the subject of many research papers that have demonstrated its positive effects. This paper appears to be an early evaluation of a CBE programme, and a discussion as to whether the true outcomes, as reflected by the small number of students, match the learning outcomes of the programme. The results from this piece of evaluatory research are mixed. I found it quite difficult to differentiate between the quantitative and qualitative elements of the results; clarity might have been better if the authors had themed the students' responses. I would also have liked to have read how the School will deal with these results or how, as a result of these results they can improve the programme and match the intended learning outcomes. I am not sure that the Take Home Messages are easily derived from the research findings.I think that I should also add that I do find it difficult to see how this paper relates to the Humanities theme

Reviewer Expertise:

NA

No decision status is available
